# Valsartan/2-Aminopyridine Co-Amorphous System: Preparation, Characterization, and Supramolecular Structure Simulation by Density Functional Theory Calculation

**DOI:** 10.3390/molecules29225467

**Published:** 2024-11-20

**Authors:** Linjie Wang, Chunan Du, Yang Yang, Pengtu Zhang, Shiling Yuan

**Affiliations:** 1 School of Chemical Engineering, Shandong Institute of Petroleum and Chemical Technology, Dongying 257061, China; linjiewang1989@hotmail.com (L.W.); 2022130@sdipct.edu.cn (C.D.); yy_tsp@163.com (Y.Y.); ptzhang@sdipct.edu.cn (P.Z.); 2 School of Chemistry and Chemical Engineering, Shandong University, Jinan 250199, China

**Keywords:** co-amorphous, valsartan, DFT, supermolecule, intermolecular connectivity

## Abstract

The objective of this work was to improve the solubility and discover a stable co-amorphous form of valsartan (VAL), a BCS class-II drug, by utilizing small molecule 2-Aminopyridine (2-AP) in varying molar ratios (2:1, 1:1, and 1:2), employing a solvent evaporation technique. Additionally, by way of a density functional theory (DFT)-based computational method with commercially available software, a new approach for determining the intermolecular connectivity of multi-molecular hydrogen bonding systems was proposed. The binary systems’ features were characterized by PXRD, DSC, FTIR, and Raman spectroscopy, while the equilibrium solubility and dissolution was determined in 0.1 N HCL and water conditions to investigate the dissolution advantage of the prepared co-amorphous systems. The results demonstrated that the co-amorphous system was successfully prepared in VAL/2-AP with a 1:2 molar ratio following solvent evaporation, whereby the hydrogen bonding sites of VAL were fully occupied. Physical stability studies were carried out under dry conditions at room temperature for 6 months. Furthermore, four possible ternary systems were constructed, and their vibrational modes were simulated by DFT calculations. The calculated infrared spectra of the four configurations varied widely, with trimer 1 showing the most resemblance to the experimental spectrum of the co-amorphous 1:2 system. Additionally, co-amorphous VAL/2-AP displayed significant improvement in the solubility and dissolution study. Notably, in the 1:2 ratio, there was almost a 4.5-fold and 15.6-fold increase in VAL’s solubility in 0.1 N HCL and water environments, respectively. In conclusion, our findings highlight the potential of co-amorphous systems as a feasible approach to improving the properties and bioavailabilities of insoluble drugs. The proposed simulation method provides valuable insights into determining the supramolecular structure of multi-molecular hydrogen bonding systems, offering a novel perspective for investigating such systems.

## 1. Introduction

A large proportion of the new chemical entities (NCEs) and about 40% of marketed drugs [1] have a low aqueous solubility and belong to biopharmaceutical classification system (BCS) classes II and IV [2,3]. These substances have great pharmaceutical potential, but the low solubility is a big challenge in different stages of drug discovery and development. Various methods have been explored to improve drug solubility [1], with one effective approach being the conversion of crystalline drugs into amorphous forms, which can enhance solubility, dissolution rate, and bioavailability [3,4]. However, amorphous systems are thermodynamically unstable and lack long-range order [5], making them at risk of transforming into stable polymorphic forms during storage or manufacturing [3,6]. Several techniques such as solid dispersion [7], polymeric micelles [8], and SEDDS [9] have been explored to improve drug solubility and oral bioavailability by inhibiting crystallization [10]. However, these techniques face challenges such as polymer aging, low encapsulation efficiency, and demulsification, which can lead to phase separation and recrystallization.

Recently, a new concept of a co-amorphous (CA) system has been introduced to address these challenges [11,12,13]. The co-amorphous formulation involves combining two drugs or a drug and a small molecule excipient to enhance the dissolution rate and physical stability of the amorphous form. The possible mechanisms of improving the stability of the mixture include the formation of intermolecular hydrogen bonding, spatial rearrangement of component molecules, and entropy increase of the system, among others [14]. Currently, this technique shows great promise in developing stable amorphous forms for insoluble drugs, representing a significant step forward in drug development [11,12,15].

**Figure 1:**
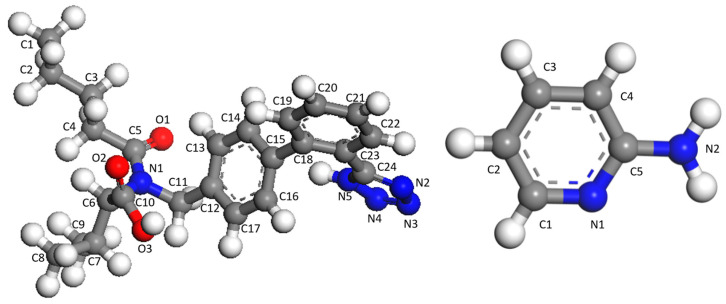
Molecular structure and atom numbering scheme of VAL and 2-AP.

Valsartan, (S)-N-valeryl-N-([2′-(1H-tetrazol-5-yl) biphenyl- 4-yl]-methyl)-valine (Figure 1), is a highly selective and competitive antagonist of the angiotensin II receptor [16]. It is widely used in the treatment of hypertension, heart failure, and post-myocardial infarction [16,17]. VAL was approved by the United States Food and Drug Administration (FDA) in 1996 and classified as a biopharmaceutical classification system (BCS) class II compound due to its poor aqueous solubility [18,19], which results in low bioavailability of approximately 23~39% in humans [20]. Various approaches, such as solid dispersions [21,22,23], nanosuspensions [24], microcapsules [25], solid state characterization of the inclusion complex of VAL with methyl β-cyclodextrin complexes [26], and recrystallization techniques [27], have been employed to improve the solubility and bioavailability of the compound. Nonetheless, the large quantities of polymer used and the thermodynamic instability of the resulting formulations have limited their applicability.

In recent years, several studies have attempted to prepare co-amorphous systems of VAL with amino acids, nifedipine, and vanillin by Huang [28], Lodagekar [29], and Ali [30], respectively. In each of these studies, VAL crystals successfully formed stable co-amorphous systems with another molecule. However, these combinations had limited effect on increasing the solubility/dissolution rate of VAL, and the ratio of co-amorphous supramolecular systems was not explored in depth. Additionally, a new crystal form F with high crystallinity has been discovered, but its solubility is similar to that of the amorphous form [31,32]. Therefore, it is crucial to prepare a new co-amorphous system to improve the pharmacokinetics of VAL.

Moreover, hydrogen bonding, as a crucial noncovalent interaction, not only influences the structure, properties, and reactivity of molecules but also exerts a profound impact on aspects such as molecular recognition, catalysis, and material design in biological systems [33]. Therefore, accurately determining the hydrogen bonding patterns in supramolecular systems is essential for understanding and uncovering their functionality and properties. However, elucidating the structures of complex systems, particularly co-amorphous systems, poses a significant challenge [34]. Infrared spectroscopy is commonly used to infer the hydrogen bonding patterns between molecules. But the limitations of infrared spectroscopy make it difficult to discern the specific mode of hydrogen bonding when multiple peaks overlap or numerous functional groups are involved in hydrogen bond formation within the structure. In this context, computational chemistry presents a new avenue for addressing such issues.

In recent years, with the rapid development of computational chemistry, quantum chemical calculations have become a powerful tool for investigating and predicting the properties of molecular systems [16,17,18]. In this study, a method aimed at obtaining accurate hydrogen bonding configurations in supramolecular systems was proposed by using a combination of experimental characterization and quantum chemical computations. Specifically, based on the compositional structure of the supramolecular system and variations observed in experimental infrared spectra, all possible hydrogen bonding arrangements were constructed. Subsequently, density functional theory (DFT) calculations were employed to simulate the infrared spectra of these arrangements and compare the computed results with experimental spectra. Ultimately, the most likely supramolecular structure of the co-amorphous system was identified to compare the simulated and experimental infrared spectra of different configurations of VAL/2-AP co-amorphous systems. To the best of our knowledge, this is the first study to determine the supramolecular structure of co-amorphous systems by combining DFT calculations and experimental infrared spectroscopy.

Compared to traditional experimental exploration methods, the approach offers several advantages. Firstly, by leveraging the high precision and accuracy of quantum chemical calculations, the hydrogen bonding interactions can be comprehensively and precisely described. Secondly, through the construction of all possible hydrogen bonding arrangements and their comparison with experimental spectra, we can more reliably determine the hydrogen bonding patterns in the supramolecular system. Lastly, this method provides a valuable foundation for further investigating the properties and functionalities of supramolecular systems. We believe that this approach will contribute to a deeper understanding of the important role played by supramolecular systems in chemistry, biology, and materials science, offering new insights and methodologies for research and applications in related fields.

The objective of this study was to improve solubility and discover a stable co-amorphous form of VAL by utilizing small molecule 2-AP in varying molar ratios (2:1, 1:1, and 1:2), employing solvent evaporation technique. 2-AP is act as a hydrogen bond donor or acceptor with VAL. The molecular interactions and crystalline properties of these combinations were characterized by PXRD, DSC, FTIR, solubility and dissolution studies, and stability studies. Additionally, a new approach combining computational modeling and infrared spectroscopic experiments was proposed for determining the intermolecular connectivity of multi-molecular hydrogen bonding system. By comparing the simulated and experimental infrared spectra obtained from different configurations of VAL/2-AP co-amorphous systems, the most probable supramolecular structure of the co-amorphous system was identified successfully. Our findings highlight the potential of co-amorphous systems as a feasible approach to improve the properties and bioavailabilities of insoluble drugs. Furthermore, the proposed simulation method provides valuable insights into determining the supramolecular structure of multi-molecular hydrogen bonding systems, offering a new perspective for investigating such systems.

## 2. Results and Discussion

### 2.1. PXRD

**Figure 2:**
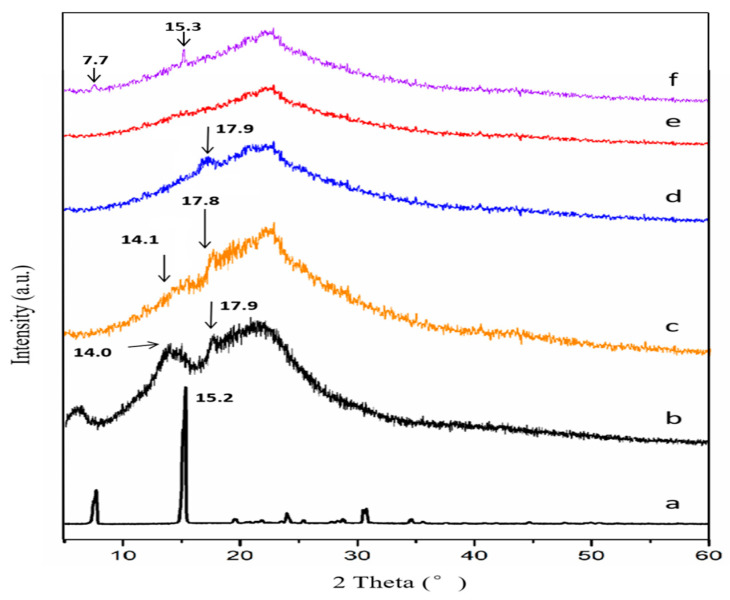
PXRD patterns of 2-AP (a), (b) VAL, (c) VAL-2-AP 2:1 CA, (d) VAL-2-AP 1:1 CA, (e) VAL-2-AP 1:2 CA, and (f) VAL-2-AP 1:3 CA.

The powder X-ray diffractograms of the VAL, 2-AP, and prepared co-amorphous systems are given in Figure 2.

The high energy barrier to conformational exchange about the amide bond and the flexibility of the alkyl chain present in VAL impedes the formation of an ordered crystal lattice, which results in substances with a lower degree of crystallinity (Figure 2b) being marketed [31]. We also utilized Jade 5 software to fit and calculate the crystallinity of valsartan, with the results indicating a crystallinity of 36.08% (Residual Error of Fit = 4.19%). The crystalline form of 2-AP showed distinct characteristic peaks at 7.7° and 15.2° in the PXRD pattern, indicating its crystalline nature as an independent excipient. After preparation, all sample ratios seemed to undergo a transformation into the amorphous form, resulting in increasingly smooth PXRD curves compared to the pure drug. However, it is important to note that this phenomenon does not necessarily indicate the successful preparation of co-amorphous samples at all four ratios. In the ratios of 2:1 and 1:1, the diffractogram displayed sharper amorphous halos at 14.1°, 17.9°, and 17.8° compared to the 1:2 pattern, indicating a notable excess amount of VAL in the two combinations. To ascertain the true stoichiometric ratio for the co-amorphous formulation, we have prepared a sample with a VAL/2-AP 1:3 ratio. In the sample prepared at a 1:3 ratio (Figure 2f), PXRD pattern revealed two distinct peaks at 7.7° and 15.3°, which correspond to the characteristic peaks of 2-AP. This indicates an excess of 2-AP in the co-amorphous sample prepared at this ratio. Therefore, the lack of significant peaks in the sample prepared with a VAL/2-AP 1:2 ratio implies that this proportion is considered the optimal reaction ratio in this system.

### 2.2. FT-IR Spectroscopy

**Figure 3:**
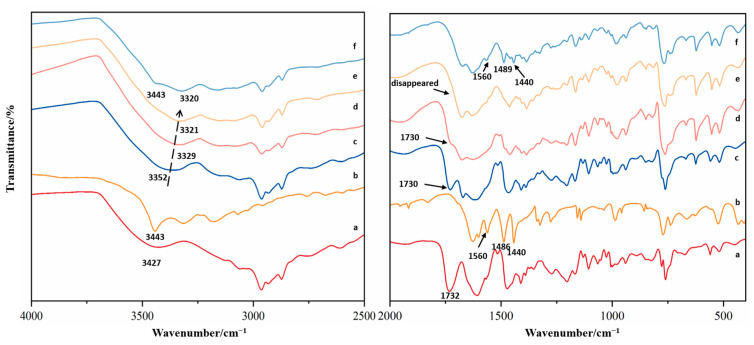
FT-IR spectra of (a) VAL, (b) 2-AP, (c) VAL-2-AP 2:1 CA, (d) VAL-2-AP 1:1 CA, (e) VAL-2-AP 1:2 CA, and (f) VAL-2-AP 1:3 CA.

To detect the interactions at the molecule level between VAL and 2-AP, the spectra of each drug individually were compared to those of the binary systems (Figure 3).

The pure VAL sample (Figure 3a) exhibited principal absorption peaks at 3427 cm^−1^, which arise from the N-H stretching of the amide group and the O-H vibration of the carboxylic acid moiety. Strong peaks at 1732 cm^−1^ and 1608 cm^−1^ were also observed, which can be attributed to the carbonyl stretch (C=O) of carboxylic acid and the amido group, respectively. In the crystalline 2-AP spectrum (Figure 3b), a relatively intense vibration band appeared at 3443 cm^−1^, which can be assigned to the NH group stretching of the amino group. Another strong peak (1627 cm^−1^) was observed in the IR spectrum and attributed to N-H vibration. Interestingly, significant shifting of absorption bands was observed in the IR spectrum of binary systems as compared to pure drugs. In the high wavenumber region, the NH and OH vibrations of VAL were shifted from 3427 cm^−1^ to 3352 cm^−1^ (Figure 3c), 3329 cm^−1^ (Figure 3d), and 3321 cm^−1^ (Figure 3e). Such shifts might be due to the intermolecular hydrogen bond interactions between the drug and excipient. In the low wavenumber region, the FT-IR spectrum of 2:1 and 1:1 co-amorphous systems (Figure 3c,d) displayed an absorption peak at 1730 cm^−1^, similar to the absorption of carbonyl group (C=O) of VAL. Remarkably, with the addition of 2-AP, the binary systems showed a gradual disappearance of the 1730 cm^−1^ peak, as indicated by the arrow. In the 1:2 ratio, the 1730 cm^−1^ peak disappeared completely, indicating that the hydrogen bonding sites of VAL were fully occupied. Furthermore, the infrared spectra of the VAL-2-AP 1:3 sample (Figure 3f) showed that compared to the spectrum of VAL-2-AP 1:2 system, the infrared spectrum of 1:3 ratio sample has obvious 2-AP characteristic peaks, such as at 3443 cm^−1^, 1489 cm^−1^, and 1440 cm^−1^. This indicates that there was an excess of 2-AP in the VAL-2-AP 1:3 ratio. Therefore, the optimal reaction ratio was found to be 1:2 of VAL and 2-AP. Moreover, in this region, the carbonyl absorption of VAL and amino absorption of 2-AP shifted and overlapped from 1730 cm^−1^ and 1627 cm^−1^ to 1671 cm^−1^, indicating the presence of an intermolecular hydrogen bond between VAL and 2-AP.

However, despite the characteristic shifts observed in their FT-IR spectra, providing evidence of significant intermolecular interactions between VAL and 2-AP, the overlapping and broad peaks resulting from the N-H and O-H vibrations of VAL hinder the precise determination of the functional groups involved in the formation of intermolecular hydrogen bonds. Additionally, 2-AP possesses multiple N-H groups, making it impossible to obtain the specific hydrogen bonding patterns from experimental infrared spectroscopy results. Given that the majority of pharmaceutical molecules are complex and large, we proposed a new computational chemistry method below to achieve an accurate determination of the supramolecular structure inherent in such multicomponent systems.

### 2.3. Raman Spectroscopy

**Figure 4:**
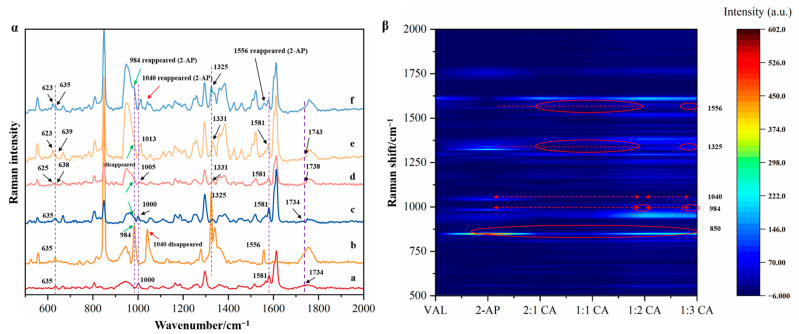
Raman spectra and contour plots of the isolated compounds and their interactions. (**α**) Raman spectra, (**β**) contour plots; (a): plain VAL, (b): 2-AP, (c): VAL-2-AP CA (2:1), (d): VAL-2-AP CA (1:1), (e): VAL-2-AP CA (1:2), and (f): VAL-2-AP CA (1:3).

Raman spectroscopy was used in an attempt to gain more insight into the molecular features of the co-amorphous samples. Figure 4 presents the experimental Raman spectra and contour plots for the isolated compounds and their interactions. The main bands, corresponding to internal modes, appear between 500 and 2000 cm^−1^. Arrows indicate spectral differences when compared with the spectra of VAL and 2-AP.

In the co-amorphous VAL-2-AP, minor shifts in the VAL bands were observed relative to neat VAL. The VAL spectrum shows a peak at approximately 635 cm^−1^ due to OCOH bending, which also appears in the 2-AP spectrum due to CCN and CCC band bending [35,36,37]. Interestingly, in the 2:1 sample, this peak remains unchanged. However, in other samples, it splits into two adjacent peaks: 625 cm^−1^ and 638 cm^−1^ in the 1:1 sample, 623 cm^−1^ and 638 cm^−1^ in the 1:2 sample, and 623 cm^−1^ and 635 cm^−1^ in the 1:3 sample. Additionally, the VAL spectrum exhibits C=O (acid) stretching and COH bending vibrations near 1734 cm^−1^. In the co-amorphous 1:2 system, this motion shifts to around 1743 cm^−1^, indicating intermolecular interactions between the carboxylic acid group of valsartan and the pyridine ring of 2-AP. The band at 1000 cm^−1^ is attributed to the NNN bending and NN stretching vibration of VAL. Upon preparing co-amorphous samples, the peaks shift to 1000 cm^−1^, 1005 cm^−1^, 1013 cm^−1^, and 1013 cm^−1^ for ratios of 2:1, 1:1, 1:2, and 1:3, respectively, suggesting that the five-membered N ring of VAL forms intermolecular hydrogen bonds with 2-AP, resulting in Raman absorption shifts.

The strong 2-AP peak at 984 cm^−1^, assigned to CN bond stretching and CCCC twisting vibration, gradually disappears as the 2-AP ratio increases [37]. This phenomenon parallels changes in the C=O vibration absorption peak in valsartan’s infrared spectrum, indicating that the formation of VAL-2-AP co-amorphous structures is sensitive to reactant ratios, achieving a pure co-amorphous sample at a 1:2 ratio. Notably, several spectral features from 2-AP, such as those at 984 cm^−1^, 1040 cm^−1^, 1325 cm^−1^, and 1556 cm^−1^, are absent in the 1:2 co-amorphous spectrum, but reappeared again in the 1:3 ratio (red arrows and ovals, Figure 4β). The horizontal lines at different Raman shifts are indicated by red dashed lines and arrows, and the appearance and disappearance of contour plots of samples with different proportions are highlighted by red ovals. This also shows that there is a significant excess of 2-AP in the VAL/2-AP 1:3 ratio, which is consistent with the infrared spectra results. Moreover, the peaks of C=N and C=O (amide) stretching vibration of Val at 1581 cm^−1^ remain unshifted, suggesting that the carbonyl group on the valsartan side chain does not form hydrogen bonds with 2-AP, consistent with infrared spectrum findings.

### 2.4. Thermal Analysis

**Figure 5:**
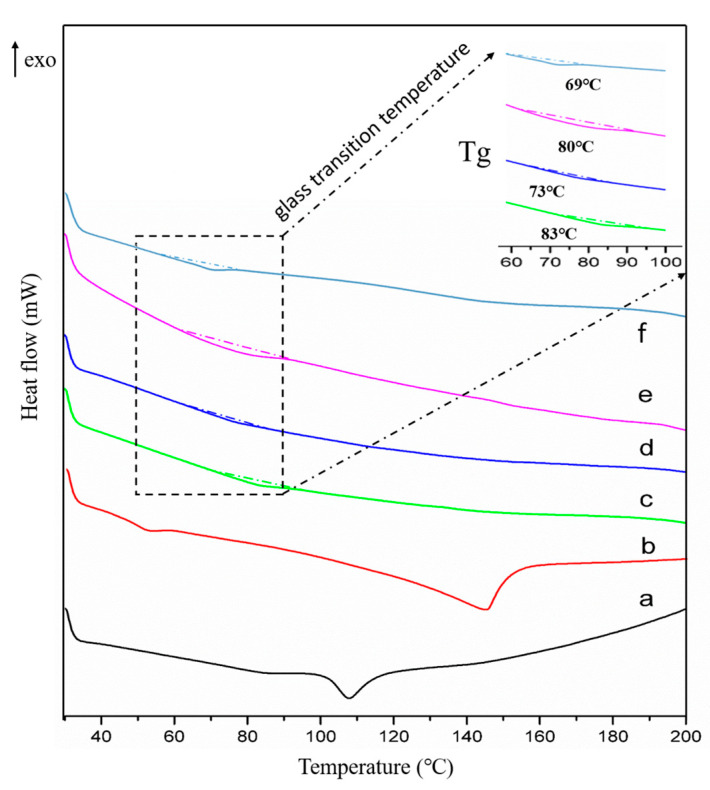
DSC thermogram of (a): plain VAL, (b): 2-AP, (c): VAL-2-AP CA (2:1), (d): VAL-2-AP CA (1:1), (e): VAL-2-AP CA (1:2), and (f): VAL-2-AP CA (1:3).

DSC thermograms (Figure 5) were obtained to investigate the thermal properties and judge the miscibility of crystalline and co-amorphous forms. In the DSC pattern of 2-AP (Figure 5b), one endothermic peak at 144 °C was observed indicating the melting point. Similarly, the DSC pattern of pure VAL exhibited a single endothermic peak at 104 °C, consistent with literature findings [38].

None of the three sample ratios displayed any melting point peaks, possibly due to the disruption of thermodynamic equilibrium in VAL after preparation. Furthermore, the glass transition (Tg) temperatures for the three systems were determined as 83 °C, 73 °C, 80 °C, and 69 °C, respectively (Figure 5c–f). Interestingly, the order of Tg values did not directly correspond to the ratio proportions, suggesting that the Tg of the binary system tended to be closer to the Tg of the component present in excess within the mixture, as reported by Allenso [28]. Notably, the Tg of amorphous VAL was found to be higher, measuring 94 °C compared to 2-AP [39]. This observation clearly indicated the presence of excess amorphous VAL in the VAL/2-AP 2:1 binary system. In the 1:1 ratio, even though VAL remains in excess, the reduced proportion of this high-Tg component leads to a significantly lower Tg of the mixture at 73 °C (Figure 5d). Conversely, in the VAL/2-AP 1:2 ratio, VAL and 2-AP fully react to form a stable co-amorphous substance, resulting in a marked increase in Tg for this system. Therefore, despite the increase in 2-AP from the 1:1 to the 1:2 ratio, the formation of the stable co-amorphous system raises the Tg of the VAL/2-AP 1:2 sample to 80 °C (Figure 5e). Furthermore, the Tg of the 1:3 ratio sample was significantly lower compared to the 1:2 sample. This could be due to the excess 2-AP in the 1:3 ratio, and since 2-AP itself has a lower Tg, it reduced the Tg temperature of the 1:2 co-amorphous system. Thus, it can be inferred that the reaction ratio played a significant role in co-amorphous formation due to the saturated nature of hydrogen bonding. Specifically, in the 1:2 (VAL/2-AP) ratio, the hydrogen bonding sites of VAL were fully occupied, which is consistent with the FT-IR and PXRD results discussed earlier.

### 2.5. Solubility Determination

**Figure 6:**
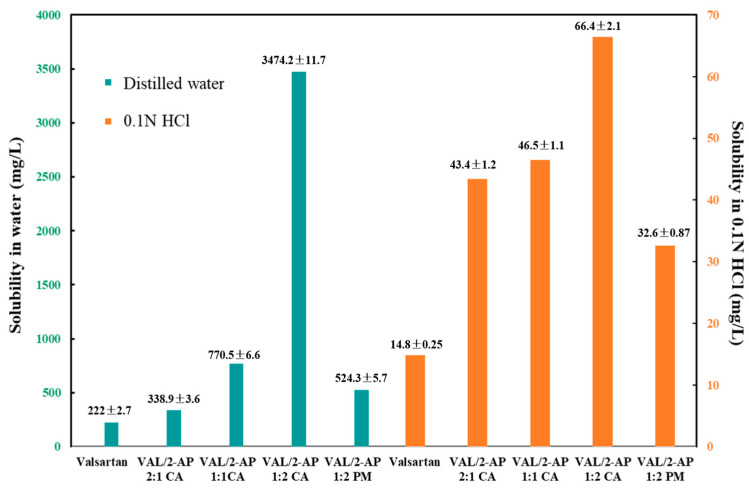
Equilibrium solubility of VAL, VAL/2-AP 1:2 physical mixture (PM), and VAL-2-AP co-amorphous systems in various media (*n* = 3, ±sd).

The equilibrium solubilities of VAL and VAL/2-AP samples in 0.1 mol/L HCl and distilled water were determined by the HPLC method, and the results are presented in Figure 6.

It was observed that the pure VAL showed a higher solubility in distilled water (222 ± 2.7 mg/L) and very low solubility of 14.8 ± 1.6 mg/L in 0.1 mol/L HCl media. In water, the solubility of the VAL/2-AP systems demonstrated significant enhancements, with values of 338.9 ± 3.6 mg/L for the 2:1 ratio, 770.5 ± 6.6 mg/L for the 1:1 ratio, and 3474.2 ± 11.7 mg/L for the 1:2 ratio. Notably, the substantial increase in solubility for the VAL/2-AP 1:2 system, accompanied by a relative standard deviation (RSD) of only 0.34%, indicates a high level of precision. This suggests that the formation of the co-amorphous state significantly enhances solubility through improved molecular interactions. Similarly, in hydrochloric acid, the solubility values for the co-amorphous systems were enhanced, yielding 43.4 ± 1.2 mg/L for the 2:1 ratio, 46.5 ± 1.1 mg/L for the 1:1 ratio, and 66.4 ± 2.1 mg/L for the 1:2 ratio. The RSD values for these measurements ranged from 2.4% for the 2:1 ratio to 3.2% for the 1:2 ratio, indicating slightly greater variability compared to the water measurements. This variability can likely be attributed to differences in solubility behavior in acidic conditions, but it also exhibits a high level of precision. In addition, the regression curve showed a good linear relationship between the mass concentration and peak area of VAL over the measurement range (r-value of 0.9951 for water and 0.9903 for 0.1 N HCl), and the average recovery of water and 0.1 N HCl medium are 99.2% (RSD = 0.96%, *n* = 9) and 98.6% (RSD = 1.31%, *n* = 9), respectively. The calibration curves established for HPLC analysis demonstrated excellent linearity, ensuring the accuracy of our solubility determinations.

From Figure 6, statistically significant improvements in the saturation solubility of the individual components compared to their pure counterparts were found in the binary amorphous systems, especially for the VAL/2-AP 1:2 system. By converting to co-amorphous systems, the solubility of VAL in 0.1 N HCL and distilled water were increased by approximately 4.5-fold and 15.6-fold, respectively, in the 1:2 ratio. These remarkable enhancements are expected to significantly enhance the dissolution and absorption of VAL in vivo. Moreover, in order to investigate the effect of the formation of the co-amorphous system on the solubility, we physically mixed VAL and 2-AP in the ratio of 1:2 for solubility measurements, which showed that the solubility of VAL and 2-AP in a 1:2 physical mixture was significantly lower at 524.3 ± 5.7 mg/L in water and 32.6 ± 0.87 mg/L in hydrochloric acid, compared to the same ratio in the co-amorphous system. This finding underscores the substantial improvement in solubility for VAL achieved through co-amorphous preparation. Furthermore, to evaluate the possibility of recrystallization, we measured the change in solubility of the co-amorphous samples over time in 0.1 N HCl and distilled water. The results demonstrated that the solubility of VAL remained stable in both solvents over the 0–48 h period, with no pronounced downward trend, indicating no significant recrystallization occurred within this timeframe.

This phenomenon might be because, after preparation, 2-AP was converted to an amorphous form with the VAL molecule by an intermolecular hydrogen bond, which was reflected by PXRD, FT-IR, and DSC studies discussed above. This binding not only enhances the interplay between the co-amorphous and the solvent but also induces changes in their stereochemistry and molecular distances. Resultantly, this binding facilitates molecular dispersion and augments the contact area between the molecule and the solvent, ultimately resulting in improved solubility. However, due to an excessive VAL, the 2:1 and 1:1 co-amorphous samples contained both the prepared VAL/2-AP co-amorphous and pure VAL, resulting in lower solubility enhancement. Thus, based on the highest solubility obtained, the optimal reaction ratio was determined to be 1:2, as mentioned previously.

### 2.6. Dissolution Test

**Figure 7:**
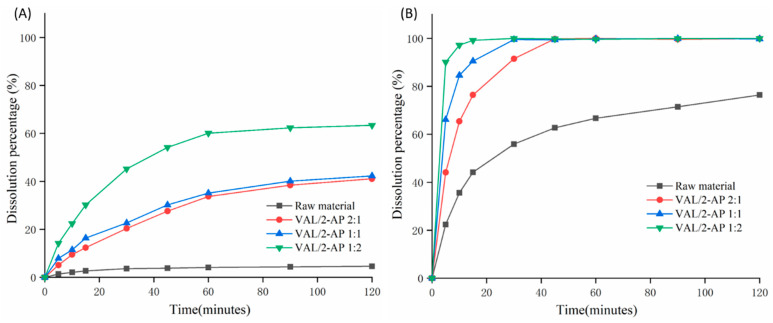
Dissolution profiles of raw drug and VAL-2-AP co-amorphous samples in pH 1.2 (**A**) and water (**B**) dissolution media.

The dissolution profiles of pure VAL and co-amorphous systems were investigated in two different dissolution media, as depicted in Figure 7. The pH-dependent solubility characteristics of the raw material led to a dissolution profile that varied with the pH of the medium. Within a span of 2 h, approximately 4.6% of the raw material was observed to dissolve at HCl medium, whereas an impressive dissolution rate of about 76.4% was achieved at distilled water condition. Notably, the impact of different reaction ratios on dissolution enhancement varies. For instance, in the case of the HCl medium, the cumulative dissolution percentage of co-amorphous systems with ratios of 2:1, 1:1, and 1:2 demonstrated significantly higher dissolution rates, measuring at 41.1%, 42.3%, and 63.4%, respectively. The preparation of co-amorphous systems greatly enhanced the dissolution of VAL in these media. These trends persisted in the distilled water dissolution medium as well, where the dissolution values of all three ratio samples increased from 76.4% to 100% following the preparation of co-amorphous systems.

Furthermore, by analyzing the first derivative of the dissolution curves (see Figure S1), it can be observed that the cumulative dissolution velocity of the VAL-2-AP co-amorphous system surpassed that of pure VAL at different stages of dissolution. This indicates a significant improvement in the dissolution performance of VAL after the preparation of the co-amorphous formulation. Among the various binary systems investigated, the VAL-2-AP 1:2 co-amorphous system exhibited the highest enhancement in dissolution.

2-AP is a highly hygroscopic small molecule drug that, upon reacting with VAL to form a co-amorphous system, undergoes hydrogen bonding and interacts with functional groups. This binding not only enhances the interaction between the co-amorphous and the solvent but also induces alterations in their stereochemistry and molecular distances. Consequently, it facilitates the dispersion of molecules and increases the contact area between the molecule and the solvent, ultimately leading to improved dissolution [40]. Furthermore, the inherent saturation of hydrogen bonds in the system affects the dissolution enhancement achieved at different reaction ratios [41]. Notably, the VAL-2-AP 1:2 co-amorphous system exhibits the highest potential for achieving hydrogen bond saturation, which aligns with the findings from infrared spectroscopy.

Therefore, after the formation of the VAL-2-AP co-amorphous system, the dissolution extent and rate of VAL were significantly increased compared to that of pure VAL. Furthermore, the dissolution enhancement observed in the 1:2 ratio system was greater than that of other systems, which is in agreement with other characterization results. Moreover, it should be noted that 2-AP exhibits some degree of toxicity. The oral LD_50_ of 2-AP in mice is 43 mg/kg, while in rats it is 200 mg/kg [42]. The recommended adult oral dose of VAL is 80 mg/day [43]. Based on the optimal molar ratio of VAL/2-AP (1:2) for preparing the co-amorphous system, the calculated dose of 2-AP is 14.2 mg/day, which is significantly lower than the LD_50_ values reported. Furthermore, the co-amorphous form has demonstrated a remarkable increase in the solubility of VAL, by 4.5-fold in 0.1 N HCL and 15.6-fold in water, which could potentially reduce the overall dose required and thereby minimize any risk associated with 2-AP. However, without comprehensive animal testing and clinical trials in humans, the toxicity and potential side effects of the co-amorphous system on the human body cannot be fully confirmed. The precise toxicological effects will still need to be validated by subsequent experiments.

### 2.7. Stability Studies

**Figure 8:**
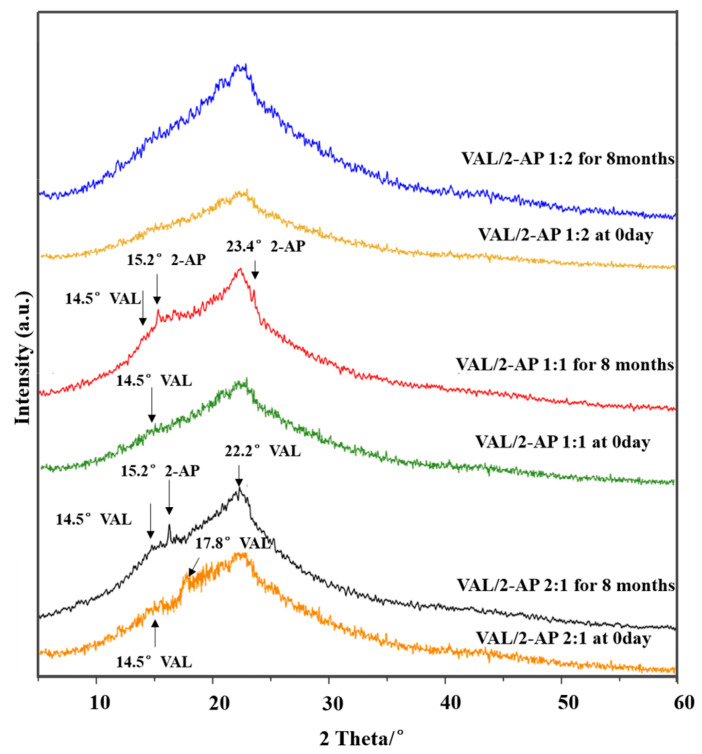
PXRD pattens of VAL-2-AP co-amorphous systems stored under dry condition at room temperature for 8 months compared to the new prepared samples.

2-AP is a highly hygroscopic compound, resulting in high moisture uptake of VAL-2-AP co-amorphous, which gradually transforms into a liquid droplet-like substance when exposed to high humidity. This causes phase separation and recrystallization under the ICH long-term stability test conditions. Therefore, due to this limitation, the stability evaluation of the co-amorphous was only performed at 0% RH condition, as a reference for the ideal state. To assess the physical stability of the amorphous sample, specifically the onset of recrystallization, it was stored for a period of 6 months under room temperature and 0% relative humidity conditions, followed by analysis using PXRD and presented in Figure 8.

In the 2:1 ratio, after 8 months of storage, the sample displayed a pronounced valsartan diffraction peak at 17.9°, and the characteristic valsartan diffraction absorption at 14.5° was also evident. In the 1:1 ratio, a characteristic absorption peak of 2-AP was observed at 15.2° and 23.4°. Furthermore, the PXRD patterns of both the 2:1 and 1:1 ratios exhibited a high degree of similarity to pure drug. This may be attributed to the insufficient saturation of 2-AP in these systems. When the content of 2-AP is low, the overall system presents itself as a mixture of pure VAL and co-amorphous VAL-2-AP. However, since pure VAL is inherently low crystallinity, the PXRD patterns appeared amorphous in all three ratios. Based on the stability results, it can be inferred that after long-term storage, the PXRD patterns of the 2:1 and 1:1 ratios indicated the separation of VAL and 2-AP, possibly due to an inappropriate feed ratio that disrupted the stability of the already formed amorphous system.

However, with an increase in the amount of 2-AP in the mixture (1:2), the stability of the eutectic combination significantly improved, and no significant recrystallization was observed even after 6 months under room temperature and 0% relative humidity conditions. This may be attributed to the gradual saturation of the hydrogen bonding sites of VAL with the addition of 2-AP, resulting in the formation of a new stable supramolecular structure that enhances the stability of the amorphous system. This finding is consistent with the FT-IR and DSC results mentioned above. Moreover, we must acknowledge that the real storage conditions may contain some humidity, and therefore it is necessary to develop formulation methods to protect the co-amorphous from moisture effects, such as combining with low hygroscopic excipients, or applying coating techniques. This will be the direction of future research.

### 2.8. Supramolecular Structure Simulation

When 2-AP molecules were introduced into VAL, they disrupted the intermolecular hydrogen bonds present in VAL and created new intermolecular interactions in the heterogeneous trimer of the VAL/2-AP co-amorphous combinations. As described above, there was a strong correlation between the saturation of the co-amorphous system and the reaction ratio, where VAL/2-AP 1:2 was the product with saturated hydrogen bond occupancy. Moreover, FT-IR analysis revealed significant intermolecular interactions between the carboxylic acid and amino group in VAL and the N-H group in 2-AP, based on their characteristic shifts.

Nevertheless, infrared spectroscopy cannot explicitly determine the mode of hydrogen bonding, particularly in a complex system with multiple molecular structures, due to peak coincidence and resolution issues [44]. This research encountered a similar issue when attempting to interpret the infrared absorption and shifts of the VAL hydroxyl group, which are implicit due to the two strong -NH vibrations. Additionally, the -NH vibration of 2-AP overlaps with that of VAL. Thus, there are multiple possibilities for the connection of the ternary system, while conforming to the results of infrared spectroscopy. To solve this problem, a new method was proposed to determine the combined structure of a multi-molecular hydrogen bonding system.

**Figure 9:**
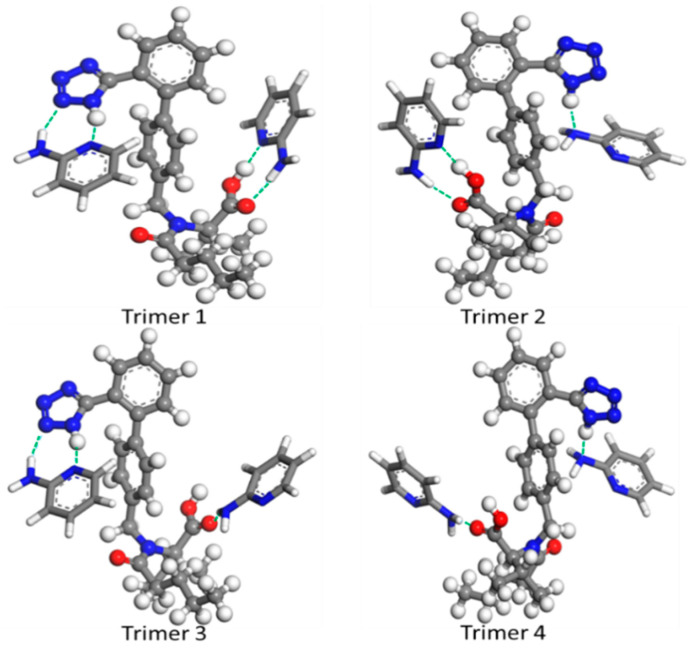
The possible configurations of the co-amorphous ternary systems after optimized by GGA/PW91.

The method consists of four steps. Firstly, the possible hydrogen bonding sites were determined by experimental infrared spectroscopy. Secondly, possible multi-molecular hydrogen bonding models were constructed in the MS program, following the hydrogen bonding rules. Thirdly, the constructed models were optimized, and the infrared spectra were calculated with the GGA-PW91 functional. Finally, the simulated and experimented spectra were compared to obtain the most likely hydrogen bonding configuration. For our research, as shown in FT-IR results, the carbonyl group on the carboxylic acid and the amino group on the five-membered ring of VAL were identified as hydrogen bonding sites. Furthermore, the hydroxyl group on the carboxylic acid group had the potential to participate in the formation of hydrogen bonds. As for 2-AP, it was challenging to identify whether the hydrogen bond site is -NH or N element in the amino group. Taken together, four possible configurations were constructed, as shown in Figure 9. The calculated wavenumbers are presented after being scaled by a scaling factor of 0.9935 [45].

**Figure 10:**
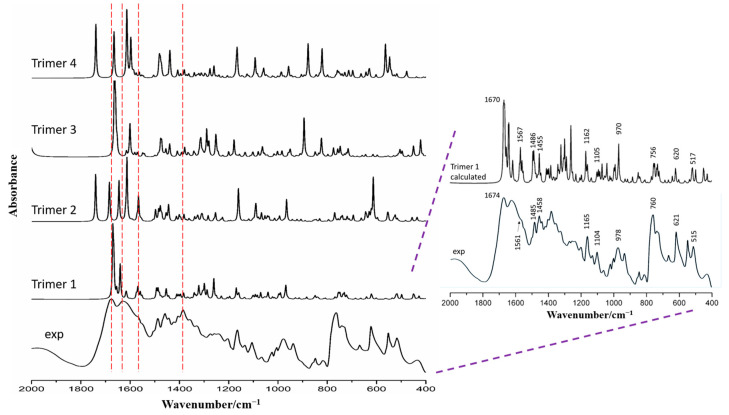
The comparison of calculated and the experimental vibrational spectrums. The red dashed line showed the position comparison of the calculated infrared spectra of different trimers relative to some diffraction peaks of the experimental spectra.

The prediction of the infrared spectrum based on DFT calculations was performed on the optimized geometry of four configurations. The calculated and experimental vibrational spectra are presented in Figure 10, and the spectral range of 2000–400 cm^−1^ was carefully examined. The peak information for all wavenumbers intervals can be found in Figure S2. Furthermore, the observed FT-IR frequencies for various modes of vibrations are compiled in Table 1 for comparison purposes.

It is evident that the infrared spectra of ternary systems calculated by the four configurations differ. The carbonyl stretching bands were calculated as 1670 cm^−1^ (trimer 1), 1739 cm^−1^ (trimer 2), 1663 cm^−1^ (trimer 3), and 1742 cm^−1^ (trimer 4); however, the band at 1674 cm^−1^ (FT-IR) was assigned to trimer 1, which reflected a similarity to its calculation. The amino and hydroxyl vibrations (Figure S2), calculated by the GGA method with PW91 functional for the four configurations, have peaks at 3338 cm^−1^ and 3229 cm^−1^ (trimer 1), 3425 cm^−1^ and 3217 cm^−1^ (trimer 2), 3330 cm^−1^ and 3626 cm^−1^ (trimer 3), 3427 cm^−1^ and 3622 cm^−1^ (trimer 4), respectively. Interestingly, the experimental result was observed at 3321 cm^−1^, which demonstrated more similarity to the trimer 1 simulation value. From the calculation and assignment of the infrared spectrum, we found an interesting business. The experimental vibration shifts from 3427 cm^−1^ in the VAL to 3321 cm^−1^ in the 1:2 co-amorphous system demonstrated the formation of the hydrogen bond. Previous research assigned this vibration to hydrogen-bonded out-of-plane N-H···N bending (Table 1). However, in DFT calculation, two strong vibration absorptions were detected at 3338 cm^−1^ and 3229 cm^−1^, which were assigned to N-H and O-H stretching modes, respectively. Interestingly, the two vibrations were exactly on both sides of the 3321 cm^−1^ peak. This might be because the low measurement accuracy of infrared spectrum overlapped the two close vibrations to a broad peak automatically. The calculated vibrational frequencies solved this problem and explained the reason why the strong O-H vibration has not appeared and discussed in previous studies. Similarly, the calculated vibration of trimer 1 at 1670 cm^−1^ was much closer to the experimental C=O vibration for the hydrogen-bonder co-amorphous VAL at 1674 cm^−1^. The vibrational modes of the molecular skeleton and benzene ring were also similar (Table 1). This finding strongly supports the assumption of a hetero-trimer formation. In the 1000–400 cm^−1^ wavelength region, valsartan exhibits characteristic absorption peaks at 760 cm^−1^ and 678 cm^−1^, corresponding to the in-plane rocking of the benzene ring and the in-plane bending of carboxylic acids, respectively. These peaks shift to 762 cm^−1^ and 622 cm^−1^ in the VAL-2-AP co-amorphous form. In the four trimers, these vibrational peaks appear at different positions: 768 cm^−1^ and 621 cm^−1^ in trimer 1, 774 cm^−1^ and 615 cm^−1^ in trimer 2, 770 cm^−1^ and 613 cm^−1^ in trimer 3, and 759 cm^−1^ and 630 cm^−1^ in trimer 4, which demonstrated more similarity to the trimer 1 simulation value. Furthermore, as shown in the blue-shaded area of Figure S2, the calculated spectrum of trimer 1 closely resembles the experimental spectrum of the VAL-2-AP co-amorphous form.

Therefore, it can be deduced from Figure 10 that trimer 1 was correctly constructed, and its simulated spectra closely resembled the experimental result. The strong and weak wavenumbers predicted by quantum mechanical calculations were comparably matched with the experimental spectra, notably the vibration absorption of functional groups after hydrogen bond formation. Correspondingly, in trimer 1 (Figure 9), one VAL and two 2-AP molecules were linked together by N–H···N (N–H from the N_5_ in VAL and pyridine-N in 2-AP), O–H···N (O– H from VAL and acceptor N from the pyridine-N of 2-AP), N–H···N (N–H from the amino in 2-AP and N4 in VAL), and N–H···O (N–H from the amino in 2-AP and O_2_ from carboxylic acid in VAL) hydrogen bonds.

Subsequently, these configurations were simulated by employing the Dmol3 module, and the corresponding interaction energies (ΔE) are tabulated in Table 2. As revealed in Table 2, the interaction energy (ΔE) of trimer 1 was −37.65 kcal/mol, significantly lower than the other three configurations. This indicated that trimer 1 is a viable and stable configuration within the VAL/2-AP 1:2 co-amorphous combination. Given the presence of the hydrogen bond acceptors of O=C and N-N and donors of O–H and N–H groups in VAL, introduction of 2-AP involving hydrogen bond acceptors of N-C and donors of N–H effortlessly impeded the hydrogen bonds in the homogeneous dimer and resulted in similar hydrogen bonds in heterogeneous combinations. From this perspective, short-range order and flexibility are the nature of co-amorphous configurations. When the VAL molecule was introduced to the crystalline 2-AP structure in a suitable reaction ratio, a plausible structure was produced in a co-amorphous combination.

To validate the accuracy and applicability of this method, we performed vibrational frequency calculations and analyses on the four configurations of the valsartan-2-AP 1:2 co-amorphous system using the hybrid-B3LYP functional. The calculated and experimental vibrational spectra are presented in Figure S3, and the observed FT-IR frequencies for various vibrational modes are compiled in Table S1 for comparison purposes. The calculated wavenumbers are presented after being scaled by a scaling factor of 0.9613 [46,47,48].

As shown in Figure S3, particularly in the blue-shaded area, the calculated spectrum obtained using hybrid-B3LYP functional for trimer 1 closely resembles the experimental infrared spectrum. Table S1 further illustrates that the vibrational frequencies of trimer 1 calculated using B3LYP are more closely aligned with the characteristic peaks observed in the experimental infrared spectrum of the co-amorphous material. For instance, the VAL-2-AP 1:2 co-amorphous sample exhibits characteristic absorption peaks at 1674 cm^−1^ and 622 cm^−1^, corresponding to the carbonyl stretching vibration and the in-plane bending of carboxylic acids, respectively. In the four trimers, these vibrational peaks appear at different positions: 1677 cm^−1^ and 613 cm^−1^ in trimer 1, 1675 cm^−1^ and 673 cm^−1^ in trimer 1733 cm^−1^ and 628 cm^−1^ in trimer 3, and 1735 cm^−1^ and 615 cm^−1^ in trimer 4, which demonstrated more similarity to the trimer 1 simulation value. Additionally, we calculated the interaction energies of the four systems using the B3LYP method, with the results listed in Table S2. The results indicate that among the four configurations, trimer 1 has the lowest interaction energy (ΔE = −24.47 kcal/mol), suggesting that trimer 1 is a viable and stable configuration within the VAL/2-AP 1:2 co-amorphous combination. This finding is consistent with the results obtained using the GGA/PW91 method.

Therefore, the new method of combining computational modeling and infrared spectroscopic experiments demonstrates high accuracy and applicability, offering significant potential for determining the supramolecular structure of multi-molecular hydrogen bonding systems.

## 3. Materials and Methods

### 3.1. Materials

Valsartan was purchased from Shanghai Dibai Biotechnology Co., Ltd. (Shanghai, China). 2-Aminopyridine and methanol anhydrous (99.8%) were obtained from Sinopharm Chemical Reagent CO., LTD. (Shanghai, China). Water was double-distilled and filtered with an Aquapro^®^ ultrapure water purification system (Dover, DE, USA). Chemicals were used as received from the companies without further purifications.

### 3.2. Preparation of Physical Mixture

VAL (1 g) and 2-AP (1 g) were individually weighed into separate 5 mL glass vials. A sufficient volume of anhydrous methanol was added to fully dissolve each sample. The two drugs were then gently stirred at 40 r/min for 24 h at 35 °C in a nitrogen-rich environment separately in their respective vials. The solutions were then rapidly evaporated to dryness under nitrogen using the cyclone-enhanced nitrogen-blowing solvent evaporator at ambient temperature. The residual solvent was thoroughly removed by subjecting the two samples to vacuum drying for 5 days at 35 °C to ensure complete solvent exclusion. After that, different molar ratios of VAL and 2-AP (1:1, 164.4 mg of VAL and 35.6 mg of 2-AP); 1:2, 139.6 mg of VAL and 60.4 mg 2-AP and 2:1, 180.4 mg of VAL and 19.6 mg of 2-AP) were precisely weighed into three vials, separately. The vial contents were stirred until a homogenous mixture was achieved, ensuring thorough mixing of the two compounds at the targeted molar composition. The resulting physical mixtures were stored in a desiccator until they were utilized in the experiment.

### 3.3. Preparation of Co-Amorphous VAL-2-AP

The co-amorphous binary blends were prepared using the cyclone-enhanced nitrogen-blowing solvent evaporation technique [49]. Around 200 mg of the mixtures were homogeneously combined in varying molar ratios of 1:1 (164.4 mg of VAL and 35.6 mg of 2-AP), 1:2 (139.6 mg of VAL and 60.4 mg 2-AP), 2:1 (180.4 mg of VAL and 19.6 mg of 2-AP) and 1:3 (121.3 mg of VAL and 78.6 mg of 2-AP) and dissolved in 10 mL of anhydrous methanol. The mixtures were gently stirred at 40 r/min for 24 h at 35 °C in a nitrogen-rich environment. The solution was then rapidly evaporated to dryness under nitrogen using the cyclone-enhanced nitrogen-blowing solvent evaporator at ambient temperature. The residual solvent was thoroughly removed by subjecting the sample to vacuum drying for 5 days at 35 °C to ensure complete solvent exclusion. The resulting precipitates were stored in a desiccator until they were utilized in the experiment.

### 3.4. PXRD

Powder X-ray diffraction was performed on a Rigaku D/max-2550PC (λ = 1.5406 Å) with Cu Kα radiation (Japan). The diffraction pattern was measured with a voltage of 40 kV and a current of 30 mA. The scanning 2θ range was 5–60° and step size of 0.02°.

### 3.5. FT-IR

FT-IR patterns were recorded using a NICOLET 380 FTIR spectrometer (Thermo Scientific, Waltham, MA, USA) in the wavelength of 4000–400 cm^−1^. Samples were prepared in KBr pellets by grinding about 1 mg of the drug with KBr and the resolution is 2 cm^−1^.

### 3.6. Raman Spectroscopy

Raman spectra were recorded by Xplora microspectrometer (Horiba Scientifics, Paris, France) via the LabSpec 6 software, which equipped with liquid N_2_-cooled CCD detector. A near-infrared laser (785 nm and ~20 mW) was used for excitation. Samples were packed in an aluminum sample holder, and spectra were collected at a resolution of 2–3 cm^−1^ with ×50 microscope objectives at room temperature. A baseline correction was applied as pretreatment with the LabSpec 6 software.

### 3.7. DSC

Thermal properties were analyzed using TGA/DSC 1 thermal analyzer (Mettler Toledo, Zurich, Switzerland). Samples (Ca. 7 mg) were placed in aluminum and heated from 30 °C to 200 °C at 10 °C /min under a nitrogen purge (50 mL/min).

### 3.8. Equilibrium Solubility

Equilibrium solubilities of crystalline and amorphous VAL and 2-AP were measured at room temperature (20 ± 0.5 °C). An excess of each powder was placed into a test tube with 0.1 N HCL (pH 1.0) and distilled water, respectively. The suspensions were mixed in a micro-oscillator at 20 °C and continuously shaken for 48 h using an SPH-200Bair bath shaker (Shiping Tech. Co., Ltd., Shanghai, China). The suspensions were filtered through a 0.45 µm syringe filter (Merck Millipore, Beary card, MA, USA) and diluted with the corresponding solution medium. The concentration of VAL was determined using HPLC for HCl and water, respectively. The experiments were conducted in triplicate, and the average solubility along with the standard deviation was reported to account for experimental uncertainty.

### 3.9. Dissolution Test

In vitro dissolution testing of VAL and co-amorphous systems was conducted in 900 mL of dissolution medium by using a USP rotating paddle apparatus at 50 rpm. The dissolution medium is composed of hydrochloric acid and distilled water, respectively, and the temperature is maintained at 37 °C. Samples, containing 40 mg of VAL, were introduced into the dissolution medium for a duration of 2 h. During this time, samples were withdrawn at predetermined intervals (0, 5, 10, 15, 30, 45, 60, 90, 120 min). For analysis, three-milliliter samples were collected, with the same volume of fresh media replacing each withdrawn sample. Subsequently, the collected samples were filtered using a 0.45 µm syringe filter (Merck Millipore, Beary card, MA, USA), followed by determination of the released drug amount through HPLC assay after suitable dilutions.

### 3.10. HPLC Analysis

The HPLC analysis was performed using a Shimadzu LC-10ADVP HPLC system (Shimadzu Technologies, Kyoto, Japan), equipped with a CAPCELL PAK C18 column (4.6 × 150 mm, 5 μm; Shiseido Fine Chemicals, Tokyo, Japan). The mobile phase consisted of a mixture of acetonitrile and water (60:40, *v*/*v*) with a pH of 2.9. The flow rate was set at 1.0 mL/min, and the detection wavelength was set at 250 nm. A calibration curve was constructed based on peak area measurements. The drug content was calculated using the following equation: (loaded drug weight/feeding drug weight) × 100. All experiments were performed in triplicates.

### 3.11. Physical Stability Study

The co-amorphous samples were stored under dry conditions at room temperature for a period of 8 months. The dry environment was maintained by employing phosphorous pentoxide. After the storage period, the samples were subjected to analysis using powder X-ray diffraction (PXRD) to ascertain the presence of any crystallization onset. In PXRD, the appearance of peaks originating from the amorphous halo in the diffractograms provided evidence of recrystallization initiation.

### 3.12. Computer Details

The initial structure of VAL and 2-AP were extracted from their crystal structure, which was obtained from the Cambridge Crystallographic Database (CCDC, Cambridge, UK) with Conquest 1.8 software (CSD, version 5.27, November 2006 plus 31 updates, Conquest version 1.8). The CCDC Codes were KIPLIG and AMPYRD for VAL and 2-AP, respectively. To determine the multi-molecular hydrogen bonding configurations, four ternary configurations were constructed by combining the experimental FT-IR spectral data with the structural information of the substance in the visualizer module. By simulating the vibrational spectra and interaction energies (ΔE) of these configurations, we can obtain an accurate description of the co-amorphous supramolecular structure formed by VAL and 2-AP.

Simulations were performed on the four ternary configurations that were constructed based on hydrogen bonding information and optimized at GGA/PW91 functional level with the DNP (Double Numerical plus Polarization) basis set and hybrid-B3LYP functional with the DNP basis set [50,51]. The frequencies were calculated at the same level of theory as geometry optimizations and the functional selection was studied and compared in our present study [52]. The convergence criteria were specified independently for maximum energy change, Max. force and Max. displacement, in which, 1 × 10^−5^ Hartree, 0.002 Hartree Å^−1^, and 0.005 Å, respectively. Vibrational frequencies and interaction energies were computed to reveal the short-range order of the co-amorphous system. All calculations were performed by using the DMol3 module in Material Studio 8.0 program package [53,54], utilizing a Dell Precision 7670 Workstation.

## 4. Conclusions

In this study, co-amorphous systems of VAL and 2-AP in different molar ratios were successfully prepared using a solvent evaporation technique. Intriguingly, with the saturation of hydrogen bonding sites, FT-IR and Raman analysis indicated that a 1:2 molar ratio was the appropriate reaction ratio for this combination, resulting in a stable and homogeneous co-amorphous system. Additionally, a new method for combining computational modeling and infrared spectroscopic experiments was also proposed for determining the supramolecular structure of multi-molecular hydrogen bonding systems. This method was successfully applied to investigate and identify molecular interactions in the co-amorphous (1:2 molar ratio) blend. The proper configuration of the ternary system was constructed and simulated, revealing the existence of hydrogen bonds of N–H···N, O–H···N, N–H···N, and N–H···O, which were well-matched with the experimentally observed spectra. The value of interaction energy indicated that the trimer had a stable configuration in the VAL/2-AP 1:2 co-amorphous system, which demonstrated a short-range order stabilized by hydrogen bonds in the ternary combinations. Furthermore, DSC and solubility studies revealed that the VAL/2-AP 1:2 co-amorphous system exhibited higher Tg and solubility, potentially resulting in increased stability and faster dissolution rates. Overall, our findings highlight the potential of co-amorphous systems as a feasible approach to improve the properties and bioavailabilities of poorly soluble drugs. The proposed simulation method provides valuable insights into determining the supramolecular structure of multi-molecular hydrogen bonding systems, offering a novel perspective for investigating such systems.

## Figures and Tables

**Table 1:** Part of observed and calculated vibrational frequencies (GGA/PBE) with their respective dominant normal modes for VAL.

Vibrational Assignment	Experimental VAL ν (cm^−1^)	Experimental Co-Amorphous ν (cm^−1^)	Calculated Trimer 1 ν (cm^−1^)	Calculated Trimer 2 ν (cm^−1^)	Calculated Trimer 3 ν (cm^−1^)	Calculated Trimer 4 ν (cm^−1^)
*ν*N_5_H_1_ *ν*O_3_H_3_	3427	3321	3338 3229	3425 3217	3330 3626	3427 3622
*ν*C_10_O_2_, δO_3_H_3_ (carboxylic acids out of phase in trimer)	1732	1674	1670	1739	1663	1742
*ρ*Benzene ring (C_18–23_H), δN_5_H_1_	1472	1485	1486	1478	1475	1478
*δ*C_8_C_9_H, C_11_H	1388	1385	1383	1381	1377	1380
*ν*C_6_–C_7_, δsC_8_C_9_H	1165	1165	1162	1162	1177	1168
In-plane bending vibration of benzene ring (C_18–23_H, C_12–17_H)	1108	1104	1105	1090	1105	1092
*δ* N_5_–H_1_	1005	978	970	968	949	956
in-plane rocking of benzene ring	761	762	768	774	770	759
*β*C_10_O_2_, δO_3_H_3_ (carboxylic acids in plane binding)	678	622	621	615	613	630

*ν*: stretching; *β*: in-plane bending; *δ*: deformation vibration; *ρ*: in-plane rocking.

**Table 2:** The energy of co-amorphous configurations of FIN/2-AP system.

Structure	Etotal (a.u.)	ΔE (kcal/mol)
VAL	−1431.47	-
2-AP	−303.65	-
Trimer 1	−2038.83	−37.65
Trimer 2	−2038.81	−25.10
Trimer 3	−2038.80	−18.83
Trimer 4	−2038.79	−12.55

## Data Availability

The original contributions presented in this study are included in the article/Supplementary Material. Further inquiries can be directed to the corresponding author.

## Supplementary Materials

The following supporting information can be downloaded at: https://www.mdpi.com/article/10.3390/molecules29225467/s1, Figure S1: Cumulated dissolution velocity curves of plain VAL and VAL/2-AP 2:1, VAL/2-AP 1:1, and VAL/2-AP 1:2 composites in pH 1.2 (A) and distilled water (B) dissolution media. Figure S2: The comparison of the experimental vibrational spectrums and the four calculated configurations by GGA/PW91: (a) experimental (b) trimer 1 (c) trimer 2 (d) trimer 3 and (e) trimer 4. Figure S3: The comparison of the experimental vibrational spectrums and the four calculated configurations by hybrid B3LYP: (a) experimental (b) trimer 1 (c) trimer 2 (d) trimer 3 and (e) trimer 4. Table S1: Part of observed and calculated vibrational frequencies (hybrid B3LYP) with their respective dominant normal modes for VAL. Table S2: The energy of co-amorphous configurations of FIN/2-AP system which simulated by hybrid B3LYP.
