# Supplementary material for: Valsartan/2-Aminopyridine Co-Amorphous System: Preparation, Characterization, and Supramolecular Structure Simulation by Density Functional Theory Calculation
Source: Molecules. 2024 Nov 20;29(22):5467. doi: 10.3390/molecules29225467 (PMC11597427; doi:10.3390/molecules29225467)
Supplement: Supplementary file 1 [file molecules-29-05467-s001.zip › molecules-3047702-supplementary.pdf]

Supporting Information

Valsartan/2-Aminopyridine Co-amorphous System: Preparation, Characterization, and Supramolecular Structure Simulation by Density Functional Theory Calculation

Linjie Wang [1], Chunan Du [1], Yang Yang [1], Pengtu Zhang [1] and Shiling Yuan [1,2,*]

[1] School of Chemical Engineering, Shandong Institute of Petroleum and Chemical Technology, Dongying 257061, Shandong Province, China
[2] School of Chemistry and Chemical Engineering, Shandong University, Jinan 250199, Shandong Province, China
* Correspondence: shilingyuan@sdu.edu.cn

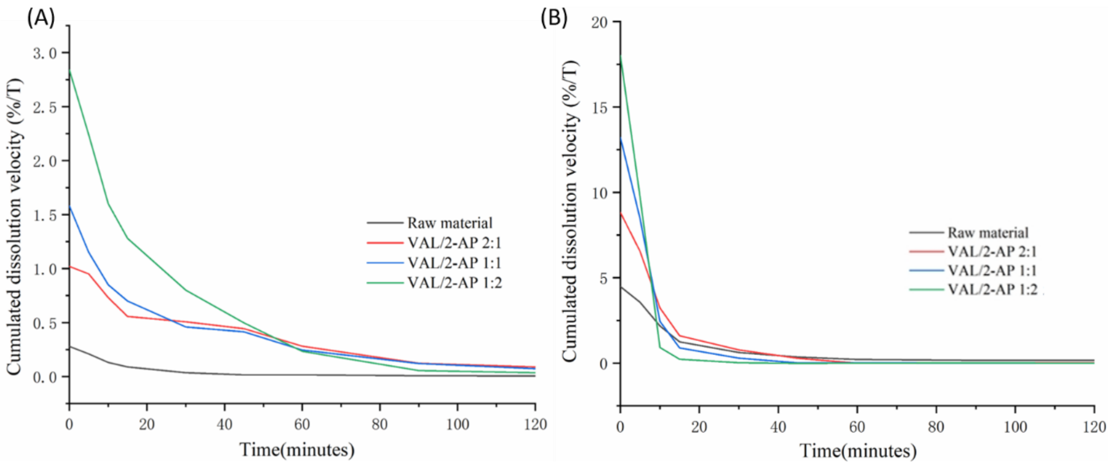

Figure S1. Cumulated dissolution velocity curves of plain VAL and VAL/2-AP 2:1, VAL/2-AP 1:1, and VAL/2-AP 1:2 composites in pH 1.2 (A) and distilled water (B) dissolution media.

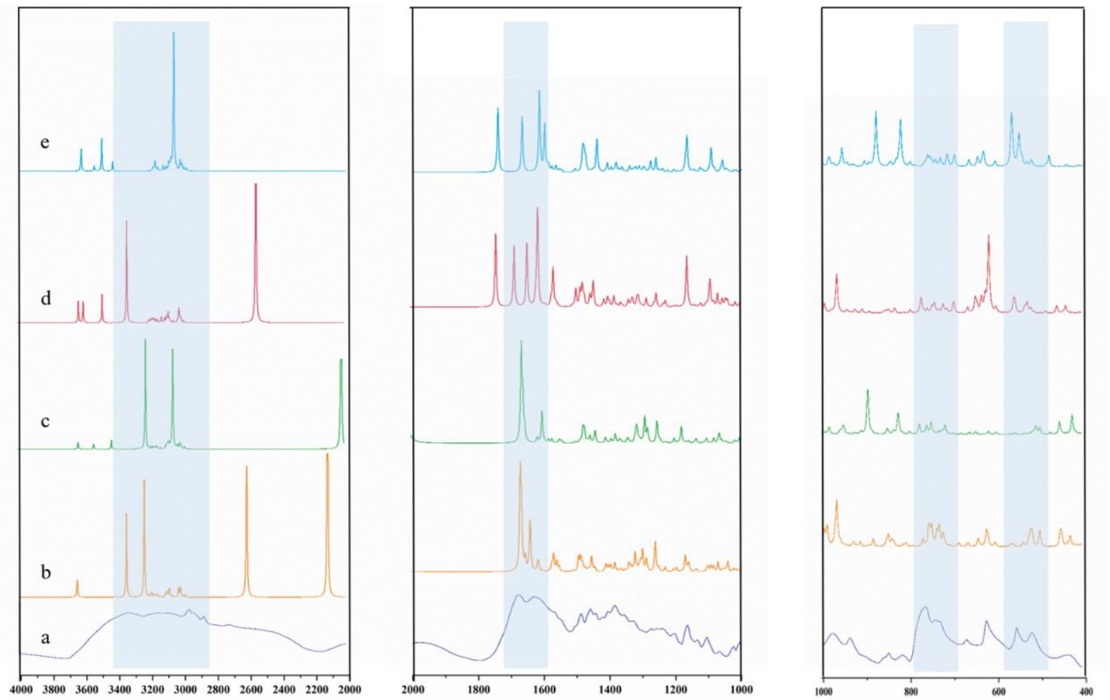

Figure S2. The comparison of the experimental vibrational spectrums and the four calculated configurations by GGA/PW91: (a) experimental (b) trimer 1 (c) trimer 2 (d) trimer 3 and (e) trimer 4.

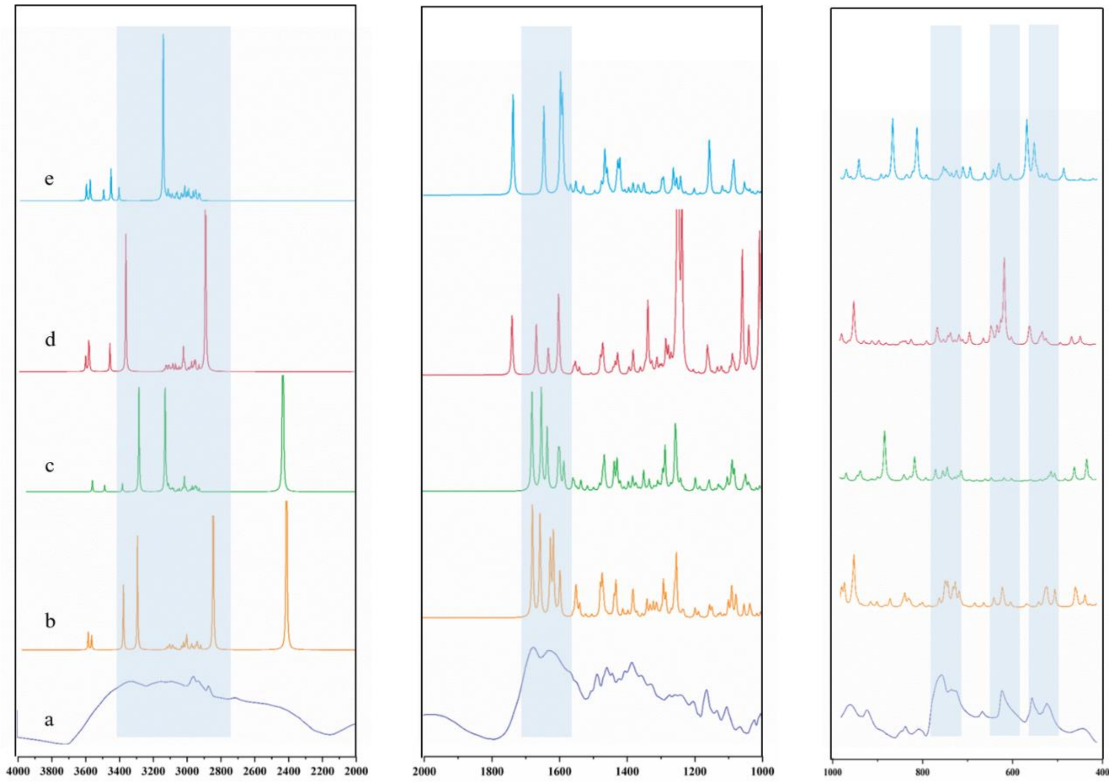

Figure S3. The comparison of the experimental vibrational spectrums and the four calculated configurations by hybrid B3LYP: (a) experimental (b) trimer 1 (c) trimer 2 (d) trimer 3 and (e) trimer 4.

Table S1. Part of observed and calculated vibrational frequencies (hybrid B3LYP) with their respective dominant normal modes for VAL.

Vibrational assignment	Experimental VAL ν (cm^{-1})	Experimental co-amorphous ν (cm^{-1})	Calculated trimer 1 ν (cm^{-1})	Calculated Trimer 2 ν (cm^{-1})	Calculated Trimer 3 ν (cm^{-1})	Calculated Trimer 4 ν (cm^{-1})
νN_5H_1	3427	3321	3339	3117	2876	3137
νO_3H_3			2406	2422	3584	3589
$\nu C_{10}O_2$, δO_3H_3 (carboxylic acids out of phase in trimer)	1732	1674	1677	1675	1733	1735
ρBenzene ring ($C_{18-23}H$), δ N_5H_1	1472	1485	1435	1433	1473	1458
δC_8C_9H, $C_{11}H$	1388	1385	1338	1345	1354	1350
νC_6-C_7, δsC_8C_9H	1165	1165	1122	1126	1128	1152
In-plane bending vibration of benzene ring ($C_{18-23}H$, $C_{12-17}H$)	1108	1104	1052	1045	1054	1053
δ N_5-H_1	1005	978	956	883	1003	981
in-plane rocking of benzene ring	761	762	751	744	738	743
$\beta C_{10}O_2$, δO_3H_3 (carboxylic acids in plane binding)	678	622	613	673	628	615

ν: stretching; β: in-plane bending; δ: deformation vibration; γ:out-of-plane bending; ρ: in-plane rocking.

Table S2. The energy of co-amorphous configurations of FIN/2-AP system which simulated by hybrid B3LYP.

Structure	Etotal (a.u.)	ΔE (kcal/mol)
VAL	-1516.841	-
2-AP	-321.692	-
Trimer 1	-2160.264	-24.47
Trimer 2	-2160.255	-18.82
Trimer 3	-2160.245	-12.55
Trimer 4	-2160.236	-6.90
